# Pre-Weaned Calf Rearing on Northern Irish Dairy Farms: Part 3—The Impact of Environmental Factors on Calf Performance

**DOI:** 10.3390/ani16152297

**Published:** 2026-07-24

**Authors:** Aaron J. Brown, Gillian Scoley, Niamh O’Connell, Alan Gordon, Steven Morrison

**Affiliations:** 1 Agri-Food and Biosciences Institute, Hillsborough BT26 6DR, Northern Ireland, UK; aaron.brown@trouwnutrition.com (A.J.B.); steven.morrison@afbini.gov.uk (S.M.); 2 Institute for Global Food Security, School of Biological Sciences, Queen’s University Belfast, Belfast BT9 5DL, Northern Ireland, UK; niamh.oconnell@qub.ac.uk; 3 Agri-Food and Biosciences Institute, Newforge Lane, Belfast BT9 5PX, Northern Ireland, UK; alan.gordon@afbini.gov.uk

**Keywords:** rearing environment, nutrition, live weight gain, feed efficiency, energy requirements

## Abstract

Nutrition, healthcare and management of the rearing environment all interact and have an impact on the performance and feed efficiency of pre-weaned dairy calves. In a survey of 54 dairy farms in Northern Ireland, average daily gain (ADG) of 369 pre-weaned calves was assessed. Dietary and nutritional information detailing intake of milk or calf milk replacer (CMR) and the composition of milk/CMR and solid feed diets was collected. Environmental and hygiene-related parameters within calf rearing facilities were recorded. It was hypothesised that suboptimal environmental management conditions would negatively affect calf performance and cause the observed average daily gain and retained energy to fall below values predicted from nutritional intake alone. Factors that tended to be negatively associated with the observed calf ADG were increased time (>50%) below lower critical temperature (LCT), increased time (>10%) above 0.4 m/s airspeed and reduced straw dry matter (<70%). Potential calf ADG was predicted using the young calf model from the *Nutrient Requirements of Dairy Cattle* (8th Edition). The predicted and observed calf ADG and retained energy (RE) were compared to assess whether the individual observed calf ADG was greater, equal to or less than that predicted by the model. Environmental factors associated with calf ADG or RE being less than the model prediction included: greater than 30 animals housed in the calf house, increased time (>10%) above 0.4 m/s airspeed and where coliforms were detected in ≤50% of milk samples on that farm. These results demonstrate the relevance of management of the rearing environment in optimising pre-weaned calf performance and feed efficiency.

## Simple Summary

Ensuring sufficient energy intake to meet demands of maintenance and targeted growth, minimising environmental stressors and optimising animal health are key elements for improving the performance of young calves during the pre-wean period. This study aimed to determine the potential achievable performance based on feeding management of pre-weaned calves on Northern Ireland dairy farms and to assess the environmental factors that may impact this performance. Calf performance was predicted using a recent calf energy and protein model along with the residual of the predicted and observed live weight gain. The impact of farm environmental factors on the residual of the predicted and observed live weight gain was evaluated to determine those that may significantly reduce the ability of calves to perform to their potential. The main environmental factors associated with a reduction in calf performance were: increased number of calves weaned per year, increased percentage of time spent below the lower critical temperature and with elevated airspeeds in the calf house, reduced bedding dry matter and the use of automatic milk feeders.

## 1. Introduction

Maximising the productive lifetime of dairy cattle is of growing importance to the dairy industry as a method of improving farm sustainability (through increasing input efficiency and financial profit) [1]. One way in which productive lifespan can be increased is through reducing the proportion of time spent in the rearing phase by reducing the age of first calving (AFC) [2]. AFC in commercial dairy herds varies but is typically shown to be in the region of 26 months in the UK [3]. By reducing AFC to 24 months, rearing costs are decreased, and an increase in milk production in the first lactation has been observed [4,5,6].

Key elements for improving the efficiency of rearing heifer replacements from birth to first calving are: (1) ensuring precise intake of digestible nutrients to meet demands of maintenance and targeted growth [7], (2) minimising environmental stressors which may reduce potential liveweight gain [8,9] and (3) optimising animal health [10]. The pre-weaned period represents an area of great opportunity to set the pace for liveweight gain and development, yet it is also a period of great risk as calves are highly susceptible to stress, morbidity and mortality [11,12,13].

A large portion of calf growth performance is driven by the retained energy from feed, after the requirement for maintenance is fulfilled [14]. In the young calf, the digestive tract is adapted for digestion of milk proteins, lactose and dietary fatty acids [15]. As such, milk or milk replacer is the calf’s voluntary preference and encompasses the majority of the diet and therefore the energy supply [16,17]. Solid feed intake increases steadily over time, stimulating the proliferation of rumen microbiota and increasing volatile fatty acid production, which aid development of the rumen epithelium, increased rumen muscularity, volume and motility [18,19]. When sufficient intake of solid feed has taken place and the rumen is developed, the calf may be successfully weaned [20].

Further to the adequate intake of digestible nutrients, efficacy of feed utilisation is reduced where animals are placed under conditions that lead to physiological stress or ill health [21,22]. Environmental temperatures below a calf’s thermoneutral zone (TNZ) increase the energy used to maintain core body temperature by adaptations such as pilo-erection, vasoconstriction or shivering [23]. Moisture in the environment exacerbates the impact of cold temperature; relative humidity (RH) above 80% has been observed to exacerbate the reduction in calf performance at sub-TNZ temperatures (8 °C) [24]. Similarly, elevated airspeeds, >0.3 m/s, reduce body insulation created by the calves’ hair coat, thus increasing the energy required to maintain body temperature [25].

Poor hygiene management is considered to have negative implications for dairy calf health and control of disease [26,27]. Increased exposure to enteric or respiratory disease-causing organisms, through poor sanitation of rearing facilities or feeding equipment, may increase incidence of scour or clinical signs of pneumonia [28,29]. In a study where 12-week-old pigs were housed in sanitised and non-sanitised pens for a period of 6 weeks, feed conversion efficiency and performance were negatively affected, whereas pneumonia prevalence was not different between the groups [30]. As hygiene management and bacterial levels vary greatly between dairy farms, the severity of exposure also differs significantly for pre-weaned calves [31]. Aside from the implications for calf disease prevalence, the impact of a heavy bacterial load on performance measures including diet-achievable growth and retained energy in calves is, to the best of the author’s knowledge, unknown.

It was hypothesised that environmental management conditions that are suboptimal for pre-wean calves would negatively affect calf performance and cause the observed average daily gain and retained energy to fall below values predicted from nutritional intake alone using existing models. The aim of this study, therefore, was to determine the potential achievable performance based on feeding management of pre-weaned calves on Northern Ireland dairy farms and to assess the environmental factors that may impact this performance. To take account of the impact of varying nutritional regimes, the objectives were to predict calf performance using a recent calf energy and protein model [32] and calculate the residual of the predicted and observed live weight gain. The final objective was to evaluate the impact of farm environmental factors on the residual of the predicted and observed live weight gain and to determine those that may significantly reduce the ability of calves to perform to their potential.

## 2. Materials and Methods

### 2.1. Data Collection

From an initial pool of seventy-five farms, representing ~3% of total dairy farm businesses in Northern Ireland, sixty-six dairy farmers agreed to participate in a survey to assess pre-weaned calf housing and management. To be eligible for inclusion, farms had to be recording for NI national benchmark figures and have a herd size of more than 60 dairy cows to ensure that adequate farm financial and physical data were available and that the rearing operation was large enough to require comparable calf rearing facilities. Farms were then stratified by size within each county, and a random sample was taken from each stratum to ensure appropriate herd size and geographical distribution [33]. Farms were visited three times; on the first visit a questionnaire was completed by the assessor, where the farm’s routine calf rearing practice was documented and calf housing was measured. Recorded calf housing measurements were dimensions of the housing structure, roof pitch, ventilation openings, calf pen area and floor slope, and material choice of roof, housing and pen walls and floors. On the second and third visits, calf liveweight was measured, giving an average daily gain (ADG) for the monitored period (14 ± 1 days).

### 2.2. Questionnaire of Farm Calf Rearing Practices

Farmers were asked the volume (litres) of colostrum provided to calves for the first feed. The number of calves reared until the typical time of weaning was ascertained. The frequency of cleaning calf pens was determined, as were whether the calf house was adjoined to other livestock buildings, and if calves shared airspace with older, post-weaned cattle (>3 months post-weaned). The maximum number of animals housed in the building was also recorded. Farmers were asked if and when calf jackets were used and at what age calves were grouped. Farms were categorised as using an automatic feeder (AMF) or teated buckets on a group or individual basis to feed milk to calves.

### 2.3. Measurements of Calf Housing and Rearing Environment

All the measures of distance were calculated using a tape measure (Stanley Tylon 5 m tape, Stanley Black & Decker, New Britain, CT, USA) or, where distances were more than ~2 m, a laser measurer (Draper LDM-40M, Draper Tools Ltd., Hampshire, UK) was used. Area (m^2^) was calculated by multiplication of length and width of the calf house, and volume (m^3^) by further multiplication of height. In cases where the housing was an irregular shape, individual section areas were cumulatively calculated to provide a total house area and volume. The area of each calf pen was calculated and the number of calves in each pen was recorded to allow determination of individual pen space allowance (m^2^/calf).

Using a predefined protocol (Cowsignals^®^ Training Company, Bergharen, The Netherlands, www.cowsignals.com), a nesting score of 1, 2, or 3, where calf legs were fully, half or not visible, respectively, was completed for each calf lying in the main calf house. On each of visits 2 and 3, samples of bedding were taken from 3 representative pens for the determination of dry matter. Each pen sample consisted of grab samples from 3 locations within the pen. Dry matter was calculated using methods described by Dunn et al. (2017) [34] and a mean value for each farm was determined.

Ambient temperature (°C), relative humidity (%) and wind speed (m/s) were logged at 10 min intervals for the duration of the study, recorded using a Kestrel 5000AG environmental meter (Kestrel Meters, Boothwyn, PA, USA). Loggers were placed between 1 and 1.5 m from the floor of the calf house. On the first farm visit, spot measurements of light were taken at calf standing height in 3 representative pens within the calf house. Measurements were taken with lights turned off and when turned on using an Extech 45170 Environmental Meter (FLIR Systems, Inc., Wilsonville, OR, USA); however, for the purposes of this study only lights-on values are discussed.

Samples of milk feed, water and concentrate feed, and swabs of feeding equipment and boot swabs were collected on visits 2 and 3 for determination of hygiene indicator organisms: total viable counts (TVCs), total coliforms (TCCs) and Escherichia Coli. A maximum of 12 samples (2 milk, 2 water, 2 concentrate, 2 boot swabs and 4 feeding equipment) were collected on each visit. Details pertaining to the collection and microbiological analysis of samples are reported in Brown et al. (2023) [35].

### 2.4. Calculation of Calf Average Daily Gain

Up to 10 pre-weaned calves in the main calf house were weighed (Tru-Test Eziweigh 5, Auckland, New Zealand) on visits 2 and 3 to enable calculation of average daily gain (kg/day). Calf age, liveweight and breed were recorded at the time of weighing. Calf breed was categorised as large dairy (Holstein, Friesian, Fleckvieh, or Montbéliarde), small dairy (Jersey or Holstein–Jersey crossbred) or beef x (Aberdeen Angus-, Hereford- or Belgian Blue-sired calves).

### 2.5. Calculation of Diet-Achievable Retained Energy and ADG

Nutritional composition (crude protein, fat, fibre and ash) of milk replacer (CMR) and calf starter concentrate feed (CS) were recorded from product labels; CMR lactose and moisture percentage were obtained from the manufacturer. For farms that fed cow’s milk to calves, milk recording information was collected to provide the milk fat and protein percentage and cow’s milk lactose content was assumed as 4.85% [32]. Farmers provided details of the quantity of liquid feed provided to calves over the first 13 weeks of life. The age of offering water, CS and forage were also recorded. Where CS was offered to calves, a given quantity was recorded by 21.2% of farms, or it was recorded as ‘ad libitum’ on the remaining farms.

Metabolisable energy (ME) of milk and CMR was calculated using equations from the *Nutrient Requirements of Dairy Cattle* [32],

ME (Mcal/kgDM) = 0.91 × (((0.945Fat% × 9.4) + (Protein% × 5.65) +(Lactose% × 4))/100)

Estimation of CS intake was completed using NASEM Equation 10-1 [32], as below

Starter DMI (g/d) = −652.525 + (BW × 14.734) + (lMEI × 18.896) +  (FPstarter × 73.303) + (FPstarter2 × 13.496) − (29.614 × FPstarter × LMEI)

where BW is calf bodyweight (kg), LMEI is the ME intake from the calf’s liquid diet (Mcal/d) and FPstarter is the time since calf starter was first offered (weeks of age). For farms that recorded quantity of starter offered, the mean difference between the recorded quantity offered and estimated intake was 380 g/day. The estimated starter intake values were used for all the calves.

Digestible energy (DE) of CS (csDE, Mcal/kgDM) was calculated as the weighted mean of the individual feed components of each CS using NASEM Table 19-1 [32]. The ME of CS (csME, Mcal/kgDM) was calculated as 0.91csDE. csME was multiplied by a factor of 0.9 in cases where LDMI (g/d) was ≥1.5% of BW to account for variation in calf early life rumen development [20,32]. Calf total daily MEI was the sum of LMEI and csMEI.

Calf energy and protein requirements were calculated using the pre-weaned calf model from the latest (8th) edition of the *Nutrient Requirements of Dairy Cattle* [32]. The equations used for calculation of the energy requirement for maintenance (MEm) and protein requirements for maintenance (MPm) are reported.

Empty bodyweight (EBW) was calculated as 0.94BW (kg) where calves were not offered CS or CS intake was predicted as 0 g/d and as 0.93BW for calves where CS intake was predicted as >0 g/d. The net energy for maintenance (NEm) required at different temperatures was calculated separately for calves aged ≤21 days and >21 days. The increase in NEm was 2.01 kcal/kgEBW^0.75^ per 1 °C below 20 °C for calves ≤ 21 days of age or 10 °C for calves > 21 days of age. The increase in NEm for individual recordings (10 min intervals) was calculated, and subsequently a daily mean increase in NEm was calculated for each day on each farm for each calf age class. Base NEm was calculated as 76.9 kcal per kgEBW^0.75^. The calf daily NEm requirement was taken as the sum of the NEm calculated for BW and the temperature-derived increase in NEm. The efficiency of ME use for maintenance (km) was taken as 0.72 for calves where CS was not offered or where CS intake was predicted as 0 g/d. Calves with predicted CS intake of >0 g/d were assigned a km of 0.69.

The efficiency of ME use for gain (kg) was calculated separately for milk/CMR and CS. For milk/CMR, kg was taken as 0.55, and the net energy for gain (NEg) from CS was calculated using Equation 10-6 [32] as

NEg (Mcal/kgDM) = (1.1376 × csME) − (0.1198 × csME^2^) + (0.0076 × csME^3^) − 1.2979

Based on the proportions of milk/CMR and CS DM in the diet, a weighted average of kg was used to calculate total diet NEg, also referred to as retained energy (RE). Diet-available RE was calculated as

RE (Mcal/d) = (Diet MEI-MEm) × kg

Empty bodyweight gain (EBG) was calculated as

EBG(kg/d) = RE/(EBW)^1/1.1^

Average daily gain (ADG, kg/d) was derived as EBG/0.94 for calves not offered CS or where CS intake was predicted as 0 g/d. Where CS intake was predicted as >0 g/d, EBG/0.91 was used [32]. Mean ADG and RE were calculated for the weighing period.

### 2.6. Calculation of Predicted Gain Protein Requirements

Dietary crude protein (CP) supply was calculated from the CP % of kg DM values from published values of the feeds. Metabolisable protein (MP) intake was calculated as a weighted average based on the proportion of LDMI and csDMI in the diet, where protein metabolism efficiency of liquid feeds was 0.95CP and liquid + CS was 0.75CP [32]. For calculation of calf metabolisable protein requirements, the MP for maintenance (MPm) and MP for gain (MPg) were separately calculated using Equations 10-10 to 10-15 from the pre-weaned calf model [32]. MPm was calculated from the MP used for scurf (hair, skin and secretions) (Scurf CP), endogenous urinary CP loss (EUCP) and metabolic faecal CP (MFP), which are calculated as follows:

Scurf CP (g/d) = 0.22 × [BW]^0.6^

EUCP (g/d) = 2.75 × [BW]^0.5^

MFP (g/d) = (11.9 × LDMI) + (20.6 × csDMI)

MPm (g/d) = EUCP + ((Scurf CP + MFP)/0.68)

Net protein required for gain (NPg) for diet-predicted gain was calculated as

NPg (g/d) = (166.2 × Diet-predicted EBG) + (6.1276 × ((Diet-predicted RE)/(Diet-predicted EBG))

Metabolisable protein requirement for gain (MPg) was then calculated as

MPg (g/d) = NPg/(efficiency of MP for gain)

where efficiency of MP for gain was calculated as

Eff of MPg = 0.70 − 0.532 × proportion of Mature BW

Mature BW was derived as 700 kg for large dairy breeds or beef crossbreeds and 520 kg for small dairy breeds (Table 21-1 and Table 21-2 from *Nutrient Requirements of Dairy Cattle* [32]). Diet-available metabolisable protein intake (MPI) was compared with the sum of calf MPm and MPg to ascertain whether diet-available protein was sufficient for diet ME-predicted gain. On a daily basis, over the observed period, 86.8 ± 2.0% of calves had insufficient NPg to support diet-predicted ADG. Therefore, diet-predicted RE was also compared with the observed RE to ensure a true comparison of energy supply. Observed RE was calculated as

Observed RE (Mcal/d) = Observed EBG^1.1^ × Observed EBW^0.205^

### 2.7. Data Management and Statistical Analysis

Calf data, farm nutrition regimes and farm environmental data were entered into Excel (Microsoft Corp., Redmond, WA, USA). Descriptive statistics were calculated to describe farm management and housing characteristics. Open responses to survey questions were categorised to provide ordinal or nominal responses; details of categorisation of responses are included in the appendix.

Five hundred and seventy-three calves were weighed on 66 farms. Calves were removed if age or liveweight gain could not be determined (n = 37). Calves older than 56 days of age (n = 109) were removed to minimise the variation in starter intake of older calves caused by differences in housing management and forage intake. Similarly, calves were removed if they were under 6 days of age on the first weighing (n = 39) to minimise variations in dietary nutrients caused by colostrum feeding. Calves that had negative growth (n = 10) were removed to minimise variation in growth potentially caused by illness, as performance models were based on calves with positive growth and do not include adjustments for the metabolic cost of disease. If only 1 or 2 calves were measured on an individual farm, these farms were removed (n = 9 calves from 6 farms) to minimise the risk of performance outliers on a particular farm. Thus, 369 calves from 54 farms were retained in models.

To determine environmental factors impacting observed calf ADG, data was analysed using a REML variance components analysis. Environmental factors were entered as a fixed effect. Calf age at the first visit (V1 Age) and bodyweight on the first visit (V1 BW), breed category and liquid DMI as % bodyweight were included as co-factors. Farm was included as a fixed effect.

The root mean standard error of prediction (RMSEP) was calculated for the predicted and observed ADG and RE values. For evaluation of farm environmental factors and calf residual ADG and RE, residual values were categorised into 3 categories (for each variable):

1. Observed > Model, where the observed ADG/RE was greater than 0 + RMSEP value.
2. Observed~Model, where the observed ADG/RE was between the positive and negative RMSEP value.
3. Observed < Model, where the observed ADG/RE was less than 0 − RMSEP value.

Data was analysed using the ‘Ordinal’ package in RStudio (RStudio v4.1.0, Boston, MA, USA). To determine the environmental factors associated with differences in residual ADG/RE, an ordinal logistic regression with random effects (proportional odds model) was performed for each. Environmental and management factors were included as fixed effects. In each model, breed category, age at visit 1 and liveweight at visit 1 were included as additional fixed effects while farm was fitted as a random effect in all the models. In each case a likelihood ratio test was used to assess the fixed effect. Models were fitted using the ‘clmm’ function, using a Laplace approximation.

## 3. Results

### 3.1. Calf Housing and Management

In total, 66 dairy farms were visited on three occasions across a period of approximately 3 months (31 January to 2 May 2019). For descriptive analysis, data from all 66 farms is included. The number of calves weaned annually on farms ranged from 16 to 250 (mean 71). In 51.5% of the calf houses, calves shared airspace with older animals (≥3 months post-weaned), and 53% of the calf houses were adjoined to other buildings. The maximum number of calves housed in calf houses ranged from 1 (individual calf hutch) to 150, and the mean was 40. This range in calf numbers is reflected in the variation in calf house volumes (Table 1).

Calves were grouped at birth (18.2% farms, n = 12), during the first week (19.7% farms, n = 13), between 1 and 4 weeks of age (39.4% farms, n = 26) or between 4 and 8 weeks of age (22.7% farms, n = 15). During the observed period, 17.6% (n = 65 calves, 22 farms) of the calves were grouped. Average nesting scores in calf pens varied from 1 to 3 (the range of the scoring system), and the mean nest score was 1.8. Calf jackets were routinely provided for young calves on 28.8% of farms (n = 19). Alternatively, jackets were only provided when temperatures were ‘cold’ (19.7%) or calves were considered small or sick (21.2%) or for either case (4.6%). AMF was used for feeding calves on 21.2% of farms. The frequency and method of cleaning calf pens are reported by Brown et al. (2023) [35]; however, in brief, for single and group pens, respectively, the most common cleaning interval was within 3 weeks (48% of farms) and within 3 and 6 weeks (43.5% of farms).

The results of feed, feed equipment and bedding hygiene (and relative hygiene targets) are reported by Brown et al. (2023) [35]. In summary, TVC was considered higher than suitable for calves in over 50% of milk/CMR, concentrate and feeding equipment samples and coliforms were detected in ~60% of milk samples, and in ~30% of concentrate and milk feeding equipment samples.

### 3.2. Farm Pre-Wean Diets

**Figure 1:**
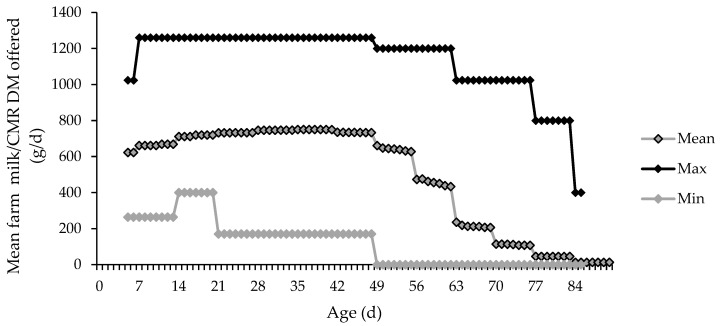
Range of milk/milk replacer DM offered to pre-wean calves on 66 dairy farms.

Cow’s milk was the sole liquid feed offered to pre-weaned calves on 18.2% (n = 12) of the farms (Table 2). On the remaining 54 farms, 25 different CMR products were used. The approximate mean cumulative DM quantity of milk/CMR offered to pre-weaned calves from 5 to 90 days of age was 41.6 kg DM/calf (range, 23.0 to 77.3 kg DM/calf) (Figure 1). Across the 66 farms, calves were completely weaned at an average of 67 days (range, 49 to 90 days of age).

### 3.3. Prediction of Calf Energy Requirements for Maintenance and Gain

The mean age and liveweight of calves sampled on visit 1 were 25 days and 51.5 kg, respectively. The observed RE, derived by the observed ADG, ranged from 0 MJ/d (calves that had 0 kg/d growth) to 13.27 MJ/d (Table 3). Mean calf liquid DMI was 0.72 kg/d, ranging from 0.19 to 1.26 kg/d. CS feed DMI varied from 0 kg/d to 1.3 kg/d, with a mean of 0.33 kg/d. Mean total MEI and CPI were 4.45 MJ/d and 243 g/d, respectively.

**Figure 2:**
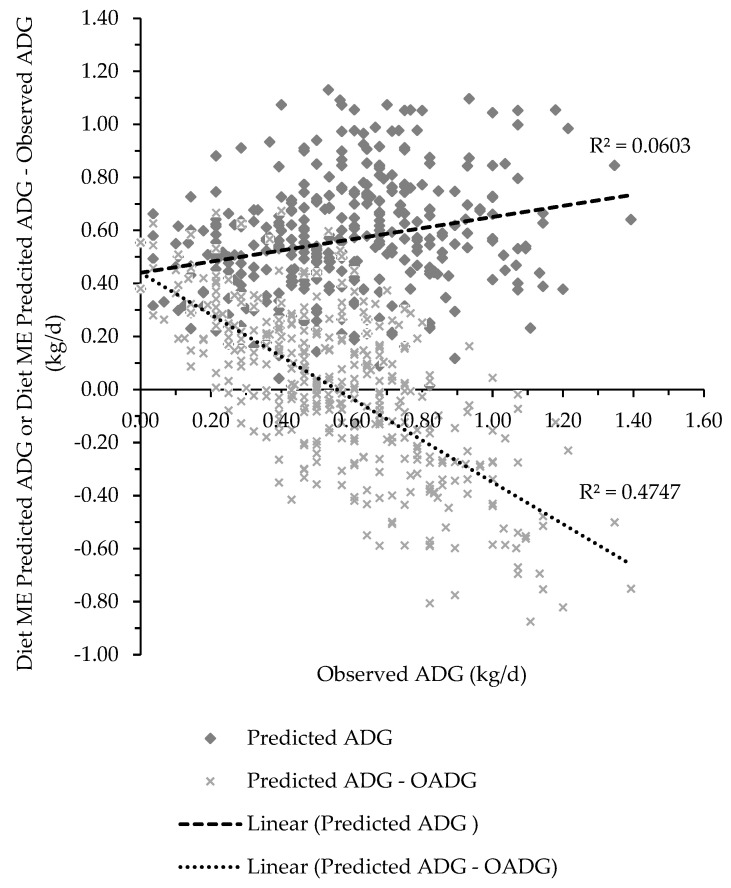
Predicted less observed values of ADG of 369 calves over the 14-day observed period.

**Figure 3:**
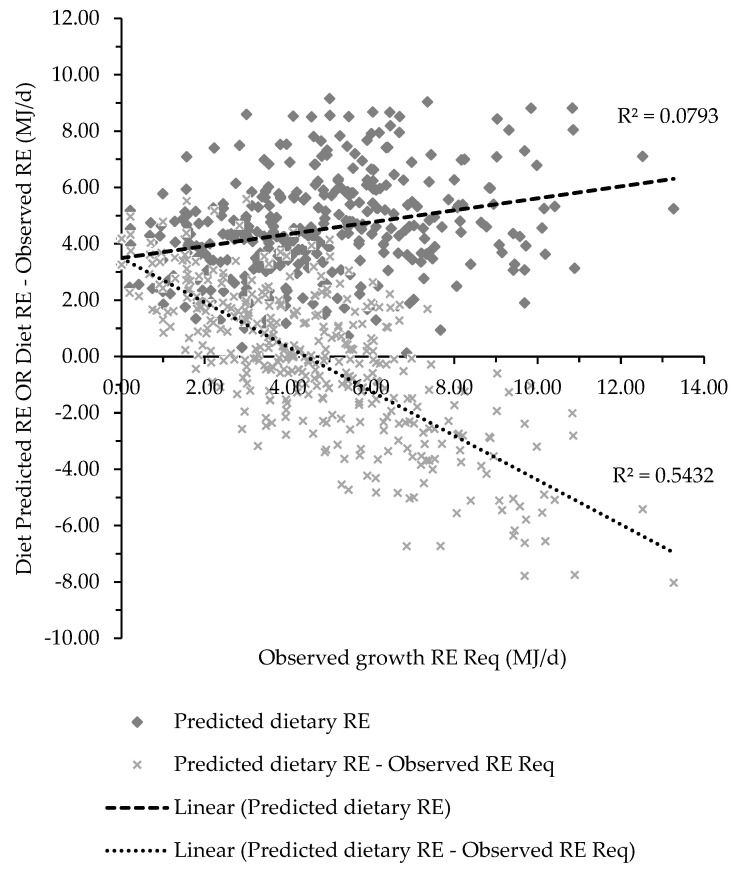
Predicted less observed values for RE of 369 calves over the 14-day observed period.

Calf maintenance requirement for energy, adjusted for environmental temperature, was 9.57 MJ/d and mean maintenance requirement for protein was 48 g/d. Efficiency of ME use for gain ranged from 0.55 to 0.42, decreasing with increased predicted proportion of CS in the diet. The mean diet-predicted RE and diet-predicted ADG were 4.49 MJ/d (Figure 2) and 0.56 kg/d (Figure 3), respectively. For calves between 5 and 30 days of age on visit 1, mean predicted ADG was 0.47 kg/d, whereas it was 0.71 kg/d for calves between 31 and 56 days of age on visit 1. Mean residual of calf ADG was −0.01 kg/day (range: +0.67 kg/d to −0.88 kg/d), whereas mean residual RE was −0.21 MJ/day (+5.59 MJ/d to −8.02 MJ/d) (Table 3).

### 3.4. Environmental Factors Related to Observed Calf ADG

Mean calf ADG across the 369 calves was 0.57 kg/d (Table 4). Mean ADG was 0.53 kg/d and 0.67 kg/d for calves aged between 5 and 30 days, and 31 and 56 days on visit 1, respectively. Calf ADG was greater on farms where 60 or fewer calves were reared to the farm’s target weaning age on an annual basis (*p* < 0.05) (0.08 kg/d greater). Calf pen cleaning frequency did not impact calf ADG, nor did TVC, coliform or *E. coli* counts of milk/CMR, starter feed, water, bedding or feeding equipment. Calf ADG was not impacted by calf housing being adjoined to other buildings, or where post-weaned animals shared airspace with pre-weaned calves (*p* > 0.1). Number of calves (>30/<30 or >50/<50) in the calf house did not impact calf ADG (*p* > 0.1). Neither calf house volume nor calf house volume per calf was associated with ADG (*p* > 0.1). Time above 80% relative humidity and average calf house light levels below 200lux were not associated with calf ADG (*p* > 0.1). However, where airspeed in the calf house was greater than 0.4 m/s for greater than 10% of the observed period, calves tended to have reduced ADG (0.46 kg/d, compared with 0.56 kg/d) (*p* < 0.1).

Higher nesting scores (mean > 1.5) were not associated with greater ADG (*p* > 0.1); however, where average bedding DM was greater than 70%, calf ADG tended to be greater, 0.58 kg/d compared to 0.51 kg/d where average bedding DM was less than 70% (*p* < 0.1). Calf ADG was not associated with greater calf space allowance (>2 m^2^/calf) (*p* > 0.1). Grouping of calves during the observed period was not associated with differences in calf performance, nor was the type of pen (group/single) that calves were housed in for the majority of the observed period (*p* > 0.1). Calves fed CMR using AMF tended to grow at a reduced rate (0.48 kg/d) than those fed using manual methods (0.57 kg/d) (*p* < 0.1). Offering of forage to calves did not impact ADG (*p* > 0.1).

Environmental factors that were significantly associated or tended to be associated with calf residual ADG (Table 5) had the same associations/trends for calf RE (Table 6). Use of AMF was associated with a lower probability of calf ADG being greater than the model prediction and a higher probability of ADG being less than the model prediction. The number of calves being weaned annually on each farm was not linked with positive or negative residual predicted–observed ADG or RE of calves. No difference in residual ADG or RE was observed where calf housing was shared with animals that were weaned for longer than 3 months, or where the building was adjoined to another building. Having greater than 30 or 50 animals in the building was not associated with residual ADG or RE. Residual ADG and RE were not affected by the use of calf jackets or by farm average nest scores. Whether calves were housed in groups or in single pens, or whether they were commingled or not during the observed period, did not relate to residual ADG or RE. Residual ADG and RE were not associated with differences between calf space allowances of ≥2 m^2^/calf or <2 m^2^/calf. Where straw bedding DM was >70%, no difference was seen in residual ADG or RE compared to DM < 70%.

Average light levels (>200/<200 lux) and percentage of time over 80% relative humidity (>50%/<50%) were not related to differences in residual ADG or RE. The probability of calves’ live weight gain being less than the model prediction tended to increase when airspeed was greater than 0.4 m/s for more than 10% of the observed period. Calf house volume and volume per calf were not associated with the probability of the observed ADG or RE being greater or less than the model prediction. Cleaning pens more or less frequently than 3 weeks or 6 weeks did not impact residual ADG or RE. Similarly, bacterial counts of bedding, water, concentrate feed and feeding equipment did not affect residual ADG or RE. However, where coliforms were detected in over 50% of milk samples, calves tended to have greater observed ADG and RE than predicted by the model.

## 4. Discussion

### 4.1. Calf Diets

Whole milk or CMR are the primary nutrient sources for young calves, as they have an efficient ability to break down and digest the inherent nutritional components. As such, calf requirements for energy, protein and minerals should be entirely met through milk/CMR provision during the first 4 weeks of life [36]. Although starter intake may commence from the first week of life, adequate intakes to support a considerable proportion of calf maintenance requirements may not take place until 1 or 2 months of age. It is recognised that, in the pursuit of encouraging starter intake to develop the rumen more rapidly, milk allowances to newborn calves have been restricted (DM < 1.5% of bodyweight, ~600 g/d) [37,38,39]. This approach was observed within this dataset, as 28.8% of farms offered a peak allowance of less than 700 g DM/d, which is meagre in comparison to other studies within the UK [40,41], and studies carried out in Norway [42] and Germany [28].

### 4.2. Calf ADG, Residual ADG and Residual RE

Calf ADG described in this study is from calves over a two-week period beginning when they were between 5 and 56 days old. The mean ADG of 0.57 kg/d is comparable to pre-weaning performance observed in calves in research studies within Northern Ireland (0.57–0.62 kg/d) [22,43], yet lower than on dairy farms in other regions: GB (0.79 kg/d) [40], Germany (0.68 kg/d) [44] and the US (0.74 kg/d) [45]. Windeyer et al. (2014) [46] observed average growth rates of 0.95 kg/d in dairy heifer calves on southwestern Ontario dairy farms, although these were average rates from birth to 3 months of age. ADG recorded in this study was over a smaller period of 14 days, and the mean age of calves at visit 1 was 25 days. However, the ADG observed is largely explained by the diets offered to calves on these farms, as the mean dietary predicted ADG was 0.56 kg/d. Diet-predicted gain for older calves (30–56 days) was greater (0.71 kg/d) than for calves of less than a month old (0.49 kg/d). The highest diet prediction for ADG in calves between 5 and 30 days old was 1.10 kg/d, suggesting that there is more scope on many farms for improved ADG within the first 4 weeks through increased nutrient intake. Increased nutritional plane in early life not only facilitates improved growth performance but has also been shown to increase metabolic activity in the ruminal epithelium and enhance maturation of the intestinal immune system, reducing the risk of enteric disease [47]. Numerous studies have demonstrated that an elevated nutritional plane in the first 2 months of life increases tissues and muscle development, leading to improved performance in lactation [5,48]. To achieve target weights at onset of puberty, and thus breeding and first calving, recommended target growth rates are at least 0.8 kg/d [4]. In this study, 316 of the 369 (85.6%) individual calf diets (assuming ad lib starter intake) were not sufficient to provide the energy requirement of 0.8 kg/d. Furthermore, 82 (22.2%) were not sufficient to provide the energy requirement of 0.4 kg/d daily gain. From these findings and based on the average ADG of calves observed in this study, it is suggested that feed allowance to pre-weaned calves on many NI dairy farms could be increased to further benefit calf growth performance.

The observed ADG and RE were less than diet-predicted ADG and RE, respectively, in calves that had ADG lower than the mean value (0.57 kg/d). Calves that performed greater than the mean value had negative residual ADG and RE (the observed gain was greater than the diet-predicted gain). This was associated with calf age at visit 1, where younger calves with lower observed ADG were more likely to have growth rates less than predicted from the diet by the model, and older calves were more likely to have ADG greater than predicted from the diet. A key limitation of the study was the prediction of starter feed intake for calves. This created the potential for variation in calf intake that was not accounted for. Greater starter intakes and subsequently variation in intake in older calves may have caused the under-prediction of ADG as calves come closer to weaning [49].

### 4.3. Environmental Factors Associated with Calf ADG, Residual ADG and Residual RE

On farms that reared a maximum of 60 calves per year, calf ADG was higher (80 g/d) than on farms where more than 60 calves per year were retained until fully weaned. Typically, as farm numbers have reduced, but herd size and milk production have increased, farms in other regions like the US have begun to separately manage heifer rearing operations [50] as labour required for calf care increases with herd size [51]. Dairy herd sizes in Northern Ireland have increased in recent years, but the average herd size in this study was 143 cows (median 124 cows), much lower than that observed in the US (367 cows) [52] and Great Britain (312 cows) [40] and unlikely to warrant a separately managed heifer rearing operation. Furthermore, 62.1% of the farms had only one person available for rearing calves, limiting available labour. Larger herd size has been associated with poorer calf health [53] and increased mortality [54,55], but to the author’s knowledge it has not been previously linked with reduced calf performance.

Automatic feeders (AMF) offer advantages to calf rearers through labour savings [56] and providing individual animal information for early detection of disease [57]. Furthermore, the ability to increase milk allowance through increased number of feeds per day [57] and to utilise gradual weaning programmes makes them an attractive proposition for dairy producers [58]. Within the current study, calf ADG tended to be lower in AMF systems than in manual feeding systems. Similarly, calves fed using AMF tended to have a higher probability of growth rates being less than the model diet prediction and a lower probability of having growth rates greater than the model prediction. Approximately one fifth (21.2%) of the farms utilised AMF, and as such, the number of calves within the study that were being fed using AMF was also proportionally low (17.6%), increasing the risk of performance outliers. No differences in calf performance have been observed between automatic and manual feeding methods in previous studies [51,56,59]. Furthermore, farmer perceptions were that switching from manual feeding systems to AMF would increase calf growth performance [60]. In the aforementioned survey, farmers who retained manual feeding systems had reservations regarding hygiene management of AMF systems. However, within this survey, as described by Brown et al. (2023) [35], CMR samples from AMF mixing bowls had lower TVC than those from manual feeding systems or in whole milk. Svensson and Liberg (2006) [61] observed greater risk of respiratory illness and reduced ADG (−0.04 kg/d) in AMF-fed calves housed in groups of 12–18 calves than in groups of 6–9 calves. Larger group sizes associated with AMF and subsequent increased risk of respiratory illness within larger groups may be related to the tendency of reduced ADG observed in the AMF-fed calves in this study.

As calves have a high ratio of body surface area to body mass, as well as the skin, subcutaneous fat layer and hair coat being thin, they are particularly susceptible to heat loss [62] and physiological stress from a variable thermal environment, the key factors being air temperature, humidity and wind speed [21]. Reduction in air temperature below the thermoneutral zone can result in behavioural changes and an increase in maintenance energy requirements as the calf makes physiological adaptations such as shivering, vasoconstriction and pilo-erection of the hair coat to maintain core body temperature [62,63]. As such, low environmental temperatures have previously been associated with reduced pre-weaned calf performance in numerous studies [40,64,65]. Within the current study, whilst not significant, a reduction in ADG was associated with calves spending over 50% of the observed period below the lower critical temperature (LCT). Reducing heat loss through interventions such as heating implements and deep bedding of straw during periods where calves are exposed to low environmental temperatures may help to mitigate negative impacts on calf performance [66,67].

The findings from the current study were suggestive (*p* = 0.072) of a reduction in calf performance due to increased exposure (>10% observed period) to elevated airspeeds (>0.4 m/s) within the calf house, otherwise referred to as draughts [25]. Similarly, and whilst unable to demonstrate a significant effect, calves subject to increased exposure to draughts tended to have lower ADG than the model prediction. Research by Bell et al. (2021) [68] found reduced calf performance (−0.19 ± 0.045 kg/d) when individually housed calves were subjected to a sub-LCT environment for >97% of the first month of life, when compared to individually housed calves housed in this environment for less than 32% of their first month of life (0.06 ± 0.34 kg/d). In that study, the proportion of time under LCT was calculated by using the effective temperature, which included adjustment for airspeed. Elevated airspeeds disrupt trapped air within the hair coat, which decreases the insulative effect and reduces the effective temperature [69]. Airspeed greater than 0.3 m/s is deemed to be a draught on young calves [70,71]; however, as the lowest range of the environmental multimeter used in this study (Kestrel 5000AG, Kestrel Meters, Boothwyn, PA, USA) was 0.4 m/s, the latter figure was used as the cut-off value. Airspeeds of this magnitude were observed for greater than 10% of the observed period on 11 farms in this study, and greater than 20% of the time on 8 farms. Increased exposure to high relative humidity (>80%) was not related to reductions in ADG. High air moisture (95% compared to 75%) has been seen to combine with low air temperature (7 °C) to reduce calf ADG [24], where temperature changes alone (15 °C compared to 7 °C) did not negatively impact ADG. As high moisture reduces the effective temperature through a wetting effect, the use of temperature–humidity index (THI) or effective temperature, where airspeed is also combined, to evaluate calf performance may prove more insightful [68]. However, in this study no difference in calf ADG was observed when calves spent more than 5% of time outside of comfortable THI (50–70) [45].

Aside from the aforementioned thermal factors, the quality of calf bedding has been seen to impact calf thermoregulation and, subsequently, growth performance. Straw bedding improved calf pre-weaned ADG by 5 to 12% when compared to wood shavings [72]. All the farms in this dataset used straw as bedding material for pre-weaned calves. The results of the current study were suggestive (*p* = 0.073) of a reduction in calf performance where bedding dry matter was less than 70%. Similarly, Quigley et al. (2017) [73] observed a trend for increased pre-weaned calf ADG (approximately 0.05 kg/d) when calves were provided with clean, dry bedding rather than soiled bedding. As calves spend a large proportion of time lying down, ensuring that bedding is dry is identified as a key element of managing cold stress, as damp bedding can increase the LCT by 5 °C when compared to dry straw [25,31,67]. Whilst not significant, the findings of previous work and the current study lend support to the advice that calf bedding should be frequently replenished to maintain higher DM, as well as reduce the risk of faecal–oral transmission of enteric pathogens [26].

Where greater than 30 animals were housed in the calf house, the probability of growth rates being less than those predicted by the model was significantly increased. Increased calf house size and volume may cause more open, unprotected calf pens; however, no differences were observed between differences in calf house total volumes and calf house volumes per calf. A larger number of calves in a single airspace may also increase the risk of virus transmission by both aerosol and direct contact, and disease has a negative impact on growth [61,74]. Previous work has highlighted the prevalence of subclinical respiratory disease, where there are no apparent visual clinical symptoms, in pre-wean calves [75]. It may be, therefore, in the present study, that where an increased number of calves were present, they were at more risk of subclinical disease, which impacted on growth rates.

Within this study, bacterial levels in feed, bedding and feeding utensils generally were not associated with differences in calf ADG or in residual ADG or RE. However, where coliforms were detected in over 50% of milk/CMR samples collected from the farm, observed calf performance tended to be more likely to be greater than that of the model, which was unexpected. The detection of coliforms may indicate a risk of disease transmission to calves, which has been shown to negatively impact calf growth; however, they are more likely to be detected in cow’s milk than milk replacer [76], which was the case in the current study [35]. Digestibility of milk replacers varies due to the variation in dairy and plant protein inclusion [77], balance of amino acids and fatty acids [78], and osmolality [79], which can impact nutrient utilisation. Additionally, within the present study, ME content of cow’s milk was an average of 4.86 MJ/kg DM higher than that of milk replacer. It is, therefore, possible that calves offered cow’s milk in the current study may have benefited from improved feed efficiency and energy content. However, given the relatively small number of farms feeding cow’s milk within the study, these findings should be interpreted with caution.

### 4.4. Study Design

The current study aims to identify key environmental factors that influence calf growth performance. Calf growth is primarily impacted by nutrient intake [65]; as such, feed intake must be included in the design of the study. As the current study was part of a survey, a variety of pre-weaned calf diets were observed. The range of calf age observed within this study also impacted on the nutrient intake proportions from liquid and solid feed, as older calves, nearer to weaning, will consume greater amounts of starter and a reduced quantity of milk/CMR [38]. Furthermore, quantity of crude protein, fat and lactose, amino acid profile, fatty acid content and digestibility of commercially available milk replacers vary, likely influencing utilisation of feed [80]. Thus, daily intake of milk/CMR, as a proportion of bodyweight, may be limited in its ability to entirely account for the dietary fraction of calf growth.

Pre-weaned calves on commercial farms are often offered starter concentrate feed and forage ad libitum [15,65,81], thus increasing the risk of variability in calf nutrient supply. Although 20% of farms in this study did not offer forage as a separate feed to pre-weaned calves, this did not negate the possibility that calves consumed bedding as a source of forage [82]. Forage intake was assumed to be negligible compared to milk/CMR and starter concentrate feed in the total nutrient intake of pre-weaned calves [15]. It was possible to predict starter intake on the basis of information provided using equations described by the National Academies of Sciences (2021) [32], but there remains some degree of uncertainty in the actual starter intakes of calves.

To assess relationships between non-dietary factors and dairy herd milk production, Bach et al. (2008) [83] offered diets identical to 47 commercial dairy herds. Within that study, milk production varied by 13 kg/d, all of which variation was attributed to non-dietary factors. To eliminate variability in farm pre-weaned calf diets, future studies that seek to evaluate the impact of commercial rearing environments on calf performance may be best served by providing participating farms with standardised pre-weaned diets.

The NASEM nutrition model used within the current study predicts requirements for calves at a specific weight and feed level, and whilst environmental conditions such as temperature can be included, the model does not account for the influence of previous management, nutrition and health status, which can impact calf growth. Future work should consider how to address periods of negative growth, which may occur as a result of ill health.

## 5. Conclusions

Pre-weaned calf ADG in NI dairy farms was typical of those seen on commercial dairy farms in other studies. Prediction of calf ADG using the provided diet plans and the most recent NASEM nutrition model yielded similar mean values. Environmental factors that were associated with reduced ADG or which tended to reduce calf ADG were: increased number of calves weaned per year on the farm, increased percentage of time spent below LCT and increased time with elevated airspeeds in the calf house, reduced bedding dry matter and the use of AMF for feeding calves. Use of AMF and increased percentage of time with elevated airspeeds were also related to reductions in calf growth in comparison to diet-predicted growth, as well as increased number of calves in the calf house. Detection of coliforms in milk samples tended to be associated with increased calf performance relative to diet-predicted performance. There is potential to improve calf growth performance through the increased provision of nutrients in the first month of life, which will ultimately also lead to benefits in health and later in life. Furthermore, management of the pre-weaned calf environment on commercial farms to reduce physiological stressors may improve calf performance and efficiency.

## Figures and Tables

**Table 1:** Descriptive summary of calf house environmental conditions on 66 dairy farms in Northern Ireland.

Environmental Factor	No. of Farms	Mean	Maximum	Minimum
Mean temperature (°C)	66	9.5	14.3	2.7
% time temperature ≤ 10 °C	66	57.1	96.6	9.1
Mean relative humidity (%)	66	82.1	92.6	70.7
% time relative humidity ≥80%	66	64.4	98.7	18.3
% time Airspeed ≥ 0.4 m/s ^1^	66	5.9	35.9	0.0
Mean Light (Lux) ^2^	66	585.0	5156.7	8.0
Calf house Volume (m^3^)	66	854.2	4878.0	37.7
Calf house Volume per calf (m^3^/calf)	66	20.6	75.3	6.2
Mean pen space allowance (m^2^/calf)	64	2.29	4.93	0.94
Mean bedding dry matter (%)	66	70.2	86.8	35.5

^1^ 0.4 m/s = lower limit of wind vane anemometer; ^2^ lights turned on.

**Table 2:** Description of milk, milk replacer and starter feed offered to calves.

Feed Type	Feed Variable (per kgDM) ^1^	No. of Farms	Mean	Maximum	Minimum
Cow’s milk	CP (%)	12	26.9	28.4	25.7
	Fat (%)	12	33.3	36.7	31.7
	ME (MJ/kgDM) ^2^	12	23.77	24.56	23.43
	ME (Mcal/kgDM) ^3^	12	5.68	5.87	5.60
Milk Replacer	CP (%)	54	23.3	26.9	20.6
	Fat (%)	54	18.7	22.9	16.5
	Lactose (%)	54	47.3	51.4	39.4
	Ash (%)	54	7.7	9.3	6.5
	ME (MJ/kgDM) ^2^	54	18.91	19.96	17.78
	ME (Mcal/kgDM)	54	4.52	4.77	4.25
Starter Feed ^1^	CP (%)	66	20.0	24.0	18.3
	Oil (%)	66	4.3	6.7	2.6
	Crude Fibre (%)	66	9.5	14.6	4.1
	ME (MJ/kgDM) ^2^	66	12.68	13.26	11.80
	ME (Mcal/kgDM)	66	3.03	3.17	2.82

^1^ Recorded from feed label; ^2^ calculated using equations from NASEM 2021, where MJ = 4.184 × Mcal; ^3^ lactose taken as 4.85 CMR.

**Table 3:** Summary of calf nutrient intake, requirements and residual ADG and RE.

	Factor	Mean	SD	Maximum	Minimum
Calf details	Visit 1 BW (kg)	51.5	10.37	82	23
	Visit 1 Age (d)	25.0	12.30	56	5
	Observed ADG (kg/d)	0.57	0.26	1.39	0.00
	Observed RE (MJ/d) ^1^	4.70	2.42	13.27	0.00
	Observed RE (Mcal/d) *	(1.12)	(0.58)	(3.17)	(0.00)
Calf Feed Intakes	LDMI (kg/d) ^2^	0.72	0.19	1.26	0.17
	csDMI (kg/d) ^3^	0.33	0.27	1.30	0
	MEI (MJ/d)	18.62	4.60	34.02	8.37
	MEI (Mcal/d) *	(4.45)	(1.10)	(8.13)	(2.00)
	CPI (g/d) ^4^	243	65.5	437	106
Calf Maintenance Requirements	MEm (MJ/d) ^5^	9.57	1.42	13.93	5.24
	MEm (Mcal/d) *	(2.29)	(0.34)	(3.33)	(1.25)
	MPm (g/d) ^6^	48	11.3	84	26
Energy and Protein available for gain	Diet weighted kg ^7^	0.50	0.03	0.55	0.42
	Predicted RE (MJ/d) ^8^	4.49	1.82	9.16	0.10
	Predicted RE (Mcal/d) *	(1.07)	(0.43)	(2.20)	(0.02)
	Predicted ADG (kg/d)	0.56	0.22	1.13	0.01
	Predicted NPg (g/d) ^9^	110	28	178	46
Diet-predicted and observed ADG and RE residual	Diet-predicted RE − Observed RE (MJ/d)	−0.21	2.59	5.59	−8.02
	Diet-predicted ADG − Observed ADG (kg/d)	−0.01	0.30	0.67	−0.88

^1^ Retained energy derived from observed ADG; ^2^ DMI from Milk/CMR; ^3^ starter feed DMI; ^4^ crude protein intake; ^5^ calf maintenance ME requirement; ^6^ calf maintenance MP requirement; ^7^ efficiency of ME for gain weighted based on proportion of liquid and solid feed intake; ^8^ retained energy derived from dietary MEI; ^9^ net protein for growth. * MJ = 4.184 Mcal.

**Table 4:** Environmental factors associated with observed calf ADG on dairy farms in Northern Ireland.

Factor	No. of Farms	No. of Calves	Mean Observed ADG (kg/d)	SED	*p*-Value
Calves weaned per year					
≤60	26	177	0.58	0.041	0.047
>60	27	184	0.50		
AMF used ^1^					
Yes	11	65	0.48	0.050	0.094
No	43	304	0.57		
% Time below LCT ^2^					
>50%	47	264	0.53	0.040	0.081
≤50%	18	105	0.60		
% Time Airspeed > 0.4 m/s ^3^					
>10%	9	55	0.63	0.054	0.072
<10%	45	314	0.55		
Average straw dry matter %					
≥70%	31	231	0.58	0.039	0.073
<70%	23	138	0.51		

^1^ Calves fed with an automatic milk feeder; ^2^ lower critical temperature; 20 °C for calves under 21 days old, 10 °C for calves 21 days old or greater; ^3^ calf house airspeed greater than 0.4 m/s, which was the lower limit of the wind vane anemometer.

**Table 5:** Impact of environmental factors on residual ADG of calves when predicted by the NASEM calf model.

Farm Environmental Factor	No of Calves	% Calves Observed > Model	Probability * (LCI-UCI **)	% Calves Observed < Model	Probability * (LCI-UCI **)	*p*-Value
No. of animals in building						
>30	182	12.7	0.06 (0.01–0.12)	18.1	0.10 (0.03–0.17)	0.058
<30	187	21.4	0.15 (0.04–0.26)	10.2	0.04 (0.00–0.08)	
AMF used						
Yes	65	9.2	0.03 (−0.01–0.07)	24.6	0.20 (0.00–0.39)	0.019
No	304	18.8	0.12 (0.05–0.19)	11.8	0.06 (0.01–0.10)	
% Time airspeed > 0.4 m/s						
>10%	55	3.6	0.04 (−0.01–0.08)	21.8	0.17 (−0.02–0.36)	0.077
<10%	314	19.4	0.11 (0.04–0.17)	12.7	0.06 (0.01–0.11)	
% of milk samples with coliforms						
≥50%	245	20.8	0.11 (0.04–0.19)	13.5	0.06 (0.01–0.12)	0.057
<50%	75	5.3	0.04 (−0.01–0.08)	20	0.18 (−0.01–0.37)	

* Probability of calf gain being greater than model prediction; ** lower and upper 95% confidence intervals.

**Table 6:** Impact of environmental factors on residual RE of calves when predicted by the NASEM calf model.

Farm Environmental Factor	No of Calves	% Calves ADG > Model ^1^	Probability * (LCI-UCI **)	% Calves ADG < Model ^2^	Probability * (LCI-UCI **)	*p*-Value
No. of animals in building						
>30	182	13.2	0.10 (0.03–0.16)	15.9	0.10 (0.04–0.15)	0.061
<30	187	21.9	0.18 (0.07–0.29)	10.2	0.05 (0.01–0.08)	
AMF used						
Yes	65	12.3	0.06 (0.00–0.11)	23.1	0.16 (0.02–0.30)	0.037
No	304	18.8	0.14 (0.07–0.22)	10.9	0.06 (0.02–0.10)	
% Time airspeed > 0.4 m/s						
>10%	55	5.4	0.06 (0.00–0.12)	18.2	0.15 (0.01–0.29)	0.085
<10%	314	19.7	0.14 (0.07–0.21)	12.1	0.07 (0.03–0.11)	
% of milk samples with coliforms						
≥50%	245	20.8	0.14 (0.06–0.23)	11.8	0.06 (0.02–0.11)	0.082
<50%	75	8.0	0.06 (0.00–0.13)	20.0	0.15 (0.01–0.28)	

^1^ Observed calf RE greater than NASEM model-predicted RE, ^2^ observed calf RE less than NASEM model-predicted RE; * probability of calf RE being greater than model prediction; ** lower and upper 95% confidence intervals.

## Data Availability

The data presented in this study is available upon request from the corresponding author.
